# Cellular bioenergetics is impaired in patients with chronic fatigue syndrome

**DOI:** 10.1371/journal.pone.0186802

**Published:** 2017-10-24

**Authors:** Cara Tomas, Audrey Brown, Victoria Strassheim, Joanna Elson, Julia Newton, Philip Manning

**Affiliations:** 1 Institute of Cellular Medicine, Newcastle University, Newcastle upon Tyne, United Kingdom; 2 Faculty of Medical Sciences, Newcastle University, Newcastle upon Tyne, United Kingdom; 3 Newcastle upon Tyne Hospitals, NHS Foundation Trust, Newcastle upon Tyne, United Kingdom; 4 Institute of Genetic Medicine, Newcastle University, Newcastle upon Tyne, United Kingdom; 5 Centre for Human Metabolomics, North-West University, Potchefstroom, South Africa; University of California Los Angeles, UNITED STATES

## Abstract

Chronic fatigue syndrome (CFS) is a highly debilitating disease of unknown aetiology. Abnormalities in bioenergetic function have been cited as one possible cause for CFS. Preliminary studies were performed to investigate cellular bioenergetic abnormalities in CFS patients. A series of assays were conducted using peripheral blood mononuclear cells (PBMCs) from CFS patients and healthy controls. These experiments investigated cellular patterns in oxidative phosphorylation (OXPHOS) and glycolysis. Results showed consistently lower measures of OXPHOS parameters in PBMCs taken from CFS patients compared with healthy controls. Seven key parameters of OXPHOS were calculated: basal respiration, ATP production, proton leak, maximal respiration, reserve capacity, non-mitochondrial respiration, and coupling efficiency. While many of the parameters differed between the CFS and control cohorts, maximal respiration was determined to be the key parameter in mitochondrial function to differ between CFS and control PBMCs due to the consistency of its impairment in CFS patients found throughout the study (p≤0.003). The lower maximal respiration in CFS PBMCs suggests that when the cells experience physiological stress they are less able to elevate their respiration rate to compensate for the increase in stress and are unable to fulfil cellular energy demands. The metabolic differences discovered highlight the inability of CFS patient PBMCs to fulfil cellular energetic demands both under basal conditions and when mitochondria are stressed during periods of high metabolic demand.

## Introduction

Chronic fatigue syndrome (CFS), also commonly known as Myalgic Encephalomyelitis (ME) and more recently Systemic Exercise Intolerance Disease (SEID), is a debilitating disease of unknown aetiology [1]. The central symptom of CFS is persistent fatigue that lasts a minimum of 6 months and which cannot be alleviated by rest or sleep. Other key symptoms of the disease include, but are not limited to, post-exertional malaise, unrefreshing sleep, memory and concentration problems, lymph node sensitivity, and muscle and joint pain [2, 3]. CFS in its most severe form can leave patients bed-bound for months or even years at a time. Each individual patient experiences the disease differently with differing symptom profiles and variability in the severity of individual symptoms on a day-to-day basis within each patient. Consequences of the disease include significant reductions in quality of life, social isolation, and marked functional disability [4–6].

Prevalence of the disease is difficult to determine with any certainty. Prevalence figures of between 0.1% [7] and 5% [8–10] have been cited. This ambiguity is in part due to the lack of standardised diagnostic criteria. A diagnosis of CFS is made via exclusion of a myriad of other disorders with similar symptom profiles, in the absence of known biomarkers for the disease [11]. The lack of specific biomarkers and definitive diagnostic criteria for the diagnosis of CFS has the potential to delay appropriate clinical intervention and inhibit progress in research into the disease. Further, the inability to clearly define diagnostic parameters presents the question of whether CFS is a single disease with one definitive cause, a consequence of several disease processes each with their own cause but similar symptoms, or one disease with different courses of disease such as Multiple Sclerosis.

It has been proposed that abnormalities in bioenergetic function may be the cause of the severe fatigue experienced by CFS patients. Factors including mitochondrial dysfunction, 5' adenosine monophosphate-activated protein kinase (AMPK) impairment, oxidative stress and skeletal muscle cell acidosis have all been associated with the CFS phenotype [12–14]. Abnormal energy metabolism has been identified as a key area of interest in CFS research recently with a number of studies investigating plasma and serum metabolomics showing altered metabolites in CFS and hypothesising that CFS is a hypometabolic syndrome [15–19].

Acquired mitochondrial dysfunction (e.g. post-viral infection) has also been an area of research interest [20–23]. Mitochondria act as the energy transduction centre of the cell and are responsible for the production of cellular ATP via respiration. Mitochondrial respiration can be inhibited by multiple factors ranging from cytokine changes to oxidative stress [24]. Studies into CFS have shown key indicators of mitochondrial dysfunction such as lower production of ATP [21, 22] and an impairment of the oxidative phosphorylation (OXPHOS) pathways [13, 25]. Additionally, crucial symptoms associated with CFS such as fatigue, exercise intolerance and myalgia are also shared by patients with primary mitochondrial diseases which are known to be caused by mitochondrial dysfunction resulting from either nuclear or mitochondrial DNA (mtDNA) mutations [25, 26]. Mitochondrial dysfunction has previously been suggested in a sub-set of the CFS population [13, 20]. However, due to a lack of definitive evidence across the CFS population others have suggested that such an association is not significant [23, 27, 28]. Two research groups have independently shown that clinically validated pathogenic mtDNA mutations are likely to be very rare in CFS cohorts [29, 30]. One study did not find any clinically validated mtDNA mutations at significant heteroplasmy levels, while the other found only one, a LHON (Leber hereditary optic neuropathy) mutation. Additionally, sub clinical levels of known mtDNA mutations are not different from those seen in the general population [31].

This study set out to specifically examine key parameters of mitochondrial function including the two major energy-producing pathways in the cell–glycolysis and OXPHOS. Changes in these parameters could indicate a mitochondrial basis for CFS and elucidate pathways in the aetiopathogenesis of the disease and identify potential targets for future study.

The aim of this case-control study was to detect and assess changes in mitochondrial functioning and cellular energy profiles systemically (using peripheral blood mononuclear cells (PBMCs)) from chronic fatigue syndrome patients and healthy controls. PBMCs can be relatively easily accessed via blood collections and were used to assess systemic mitochondrial function in controls and CFS patients due to their systemic exposure.

Abnormal PBMC bioenergetics have previously been investigated in other chronic diseases including obesity [32], type 2 diabetes mellitus (T2DM) [33], and rheumatoid arthritis[34]. Hartman *et al*. showed higher basal respiration and maximal respiration in T2DM patients [33]. Like patients with CFS, patients with T2DM often experience fatigue [35, 36]. Previous research has shown maximal respiration and reserve capacity in PBMCs to correlate with physical function and strength [37]. Symptoms described in this study such as muscle pain, weakness and intolerance to exercise are also common symptoms among CFS patients. Given the similarity in symptom profiles in the two diseases, in this study we aim to investigate if PBMC abnormalities in bioenergetic function also exist in CFS [38–40].

## Methods

Blood samples were obtained from patients fulfilling the Fukuda Diagnostic criteria for CFS after obtaining ethical approval from the National Research Ethics Committee North East–Newcastle & North Tyneside 2 [41]. Samples from healthy controls were collected through the Institute of Cellular Medicine (Newcastle University) blood study after obtaining ethical approval from the National Research Ethics Committee North East–County Durham & Tees Valley. Samples were gathered after informed written consent was obtained.

### PBMC Preparation

Blood samples were processed using the Histopaque® method. Briefly, the whole blood sample was centrifuged at 700 x g for 10 minutes and plasma removed. Blood was made up to its original volume with sterile PBS (Sigma Aldrich D8537). Density gradients were prepared with Histopaque® 1.077 (Sigma Aldrich 10771) and Histopaque® 1.119 (Sigma Aldrich 11191). Blood was slowly layered on top of the Histopaque® gradient and the tube spun at 700 x g for 30 minutes with the break off. Plasma layer was aspirated off and the PBMC layer collected. PBMCs were washed with fresh PBS and either used for experiments immediately or frozen at -80°C after being combined with freezing medium (40% FBS (Sigma Aldrich F0804), 10% DMSO (Sigma Aldrich D8418) and 50% RPMI-1640 (Sigma Aldrich R7388). To revive frozen samples, vials were rapidly defrosted in a water bath at 37°C and added to 10ml of fresh RPMI-1640. Cells were centrifuged at 700 x g for 10 minutes, the supernatant removed and cells resuspended in fresh RPMI-1640. Cell viability was then determined using the trypan blue method (see below). PBMC experiments were conducted using RPMI-1640 medium supplemented with 10% FBS and 1% penicillin-streptomycin (Sigma Aldrich P4333). Blood samples were processed within 4 hours of blood collection.

### Extracellular flux analysis

OXPHOS and glycolytic function of cells were determined using the Seahorse XF^e^96 extracellular flux analyser according to manufacturer’s protocols [42, 43]. The XF^e^96 extracellular flux analyser used in this study provided a high-throughput, 96-well, fully automated format for the analysis of OXPHOS and glycolysis. With the XF^e^96, mitochondrial respiration is measured by recording the rate of decrease of the concentration in oxygen in the assay medium. Probes form a transient micro-chamber within each well allowing changes in oxygen level and proton concentration to be easily detected. The rate of glycolysis of the cells was measured by recording the rate of increase in proton concentration in the assay medium, also known as the extracellular acidification rate (ECAR). Oxygen consumption rate (OCR) was used as an indicator of OXPHOS while ECAR was used as an indicator of glycolytic conversion of glucose to lactate.

**Fig 1:**
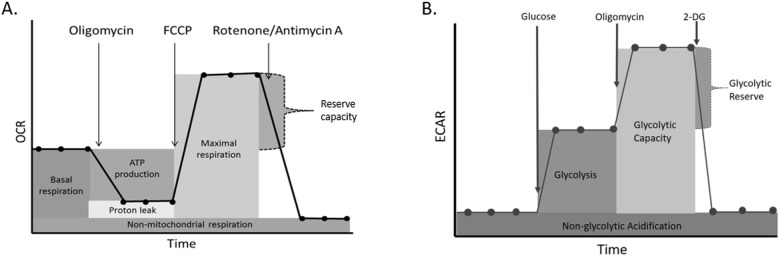
**A.** Profile of the key parameters of mitochondrial respiration measured during a mitochondrial stress test. **B.** Key parameters measured during a glycolysis stress test. Based on images found in the XF report generator user guide [44].

Cells were seeded on a microplate (Agilent Technologies 101085–004), coated with poly-D-lysine (Sigma Aldrich P7886) to aid attachment, at pre-determined cell densities (500,000 cells/well) and incubated overnight at 37°C and 5% CO_2_ prior to experiments. Each sample was seeded at least in quadruplicate to aid measurement reliability. Oxygen consumption rate (OCR) and extracellular acidification rate (ECAR) were measured following the sequential addition of test reagents. For the mitochondrial stress test these were; 1μM oligomycin (Sigma Aldrich 75351), 3μM Carbonyl cyanide-4-(trifluoromethoxy)phenylhydrazone (FCCP) (Sigma Aldrich C2920) and 0.5μM rotenone (Sigma Aldrich R8875) and antimycin A (Sigma Aldrich A8674). Basal respiration, ATP production, proton leak, maximal respiration, reserve capacity, non-mitochondrial respiration, and coupling efficiency were calculated as described by the manufacturer (Fig 1A) [44]. For the glycolysis stress test the compounds added were; 10mM glucose (Sigma Aldrich G7021), 1μM oligomycin (Sigma Aldrich 75351), and 50mM 2-deoxy-glucose (Sigma Aldrich D6134). This allowed glycolysis, glycolytic capacity, glycolytic reserve, and non-glycolytic acidification to be calculated as described by the manufacturer (Fig 1B) [44]. Reliability of reagents was ensured by using reagents aliquoted from a single batch. Data were normalised for protein concentration following a bicinchoninic acid (BCA) assay (Fisher Scientific 10741395) conducted according to manufacturer’s instructions [45]. Briefly, standards (in triplicate) and working reagent (50 parts BCA reagent A and 1 part BCA reagent B) were prepared. Medium was removed from the microplate and 25μl of ice cold cell lysis buffer (Sigma Aldrich 4719956001) added. Cells were scraped to ensure detachment from the plate. 200μl was added to each well, including standards, and the entire contents of the well transferred to a fresh 96 well plate. This was incubated at 37°C in the dark for 30 minutes before absorbance was read at 562nm. A standard curve was created using the standards and protein concentration of the samples determined. Analysis was conducted using Wave software version 2.2.0.276 and Microsoft Excel 2013.

### Trypan blue

Trypan blue (Sigma Aldrich T8154) was used to determine cell viabilities. Equal volumes of cell suspension and trypan blue were mixed and cells counted on a haemocytometer. Dead cells appear blue as they are permeable and take up the dye while living cells are impermeable to the dye and appear uncoloured. Living cells were calculated as a percentage of the total number of cells to give cell viability.

### Statistics

Groups were compared using a two-way ANOVA with least significant difference (LSD) test and post-hoc Bonferroni correction for multiple comparisons were used where stated. Two-way ANOVAs were carried out using IBM SPSS Statistics 22. All other statistical analysis was conducted using MiniTab 17 statistical software. The Anderson-Darling test for normality was used to ensure normally distributed data before Student’s t-tests were carried out after assessing data to confirm equal variances. Pearsons correlations were used to determine correlation statistics. Graphs were created using Graphpad Prism 7.

## Results

### Age and sex of participants are shown in Table 1.

**Table 1:** Age and gender composition of patient and control cohorts.

	Control	CFS
**Total participants**	35	52
**Age (mean±SD)**	36.6±12.0	42.8±13.7
**Female/male ratio**	27/8	44/8

Initial experiments were carried out in order to establish optimal cell number, oligomycin and FCCP concentrations as per the manufacturer’s instructions (S1 File) [46].

### Effect of freezing on PBMC OCR

**Fig 2:**
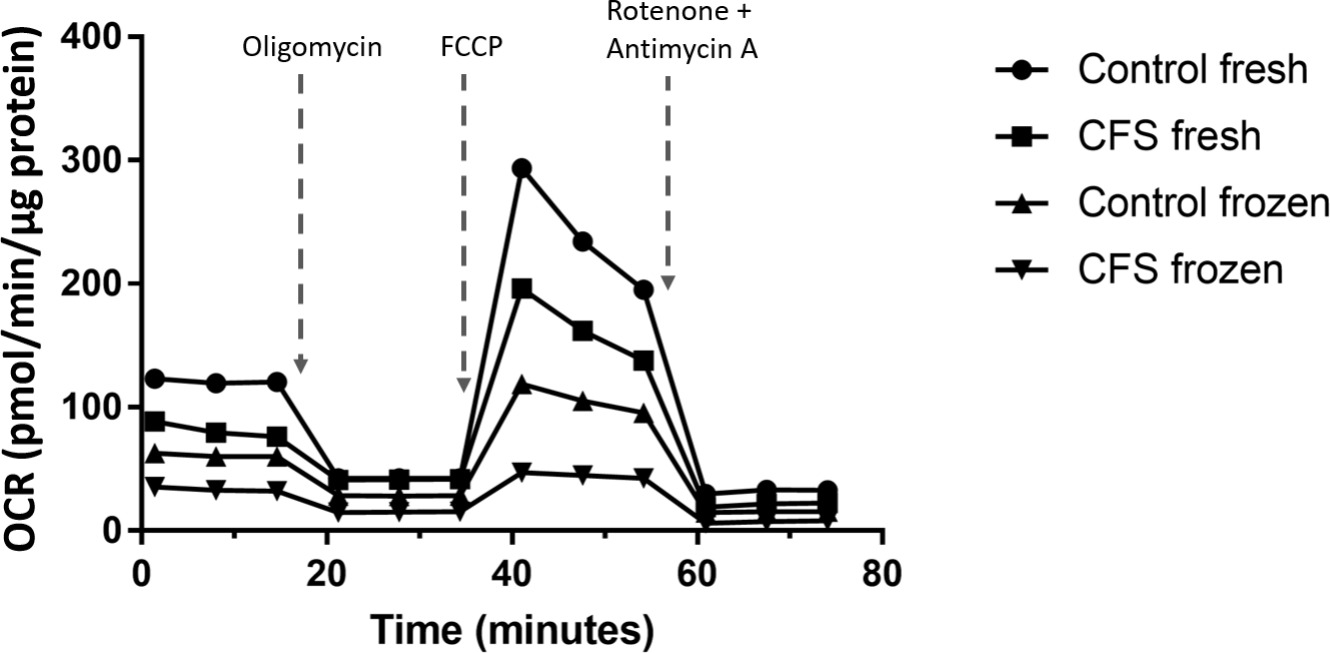
Example extracellular flux traces of a mitochondrial stress test performed on fresh and frozen PBMCs isolated from CFS patients and controls.

Blood sample collection from CFS patients was sporadic and unpredictable, therefore, it was deemed necessary to freeze samples so they could be run at the same time to negate inter-plate variation. For this reason, mitochondrial stress tests were performed on freshly isolated PBMCs and those that had been frozen at -80°C on the day of collection and subsequently revived in order to determine the effect of freezing on mitochondrial function which has previously been considered with conflicting results [47, 48]. These experiments were conducted with cells incubated in high (10mM) glucose medium. Example traces from the mitochondrial stress test are shown in Fig 2.

**Fig 3:**
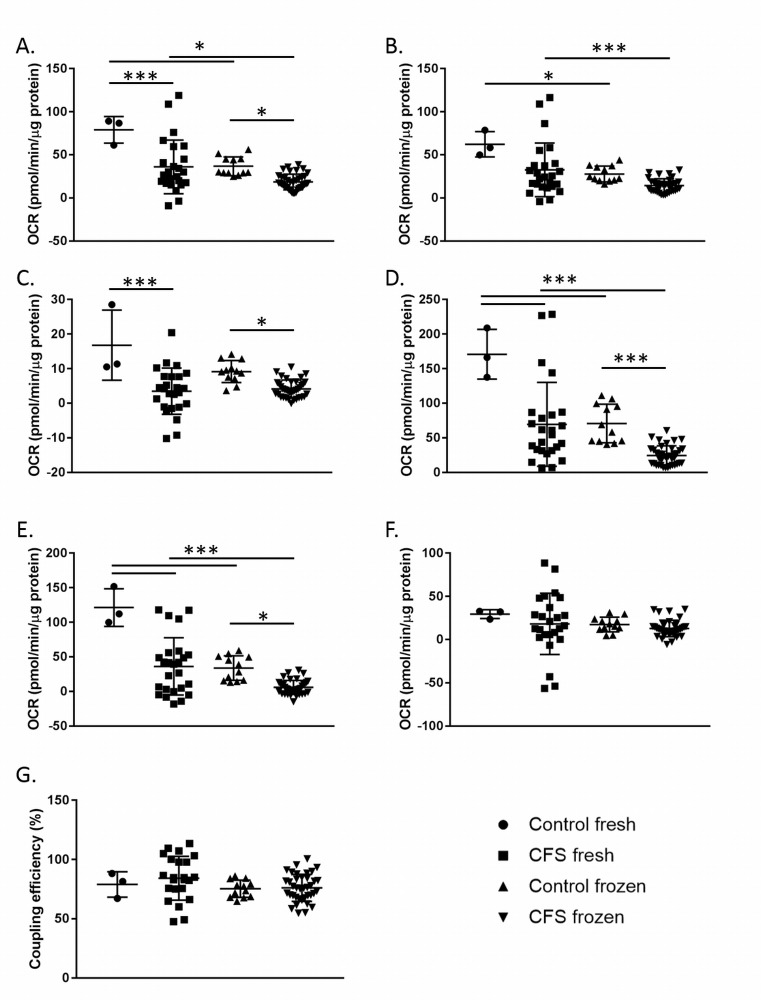
Results from a mitochondrial stress test conducted using fresh and frozen CFS and control PBMCs. **A.** Basal respiration. **B.** ATP production. **C.** Proton leak. **D.** Maximal respiration. **E.** Reserve capacity. **F.** Non-mitochondrial respiration. **G.** Coupling efficiency. Control fresh n = 3; CFS fresh n = 25; Control frozen n = 12; CFS frozen n = 38. * denotes p ≤ 0.05; *** denotes p ≤ 0.005. Groups were compared using a two-way ANOVA with LSD test and post-hoc Bonferroni correction for multiple comparisons.

The seven parameters of respiration (basal respiration, ATP production, proton leak, maximal respiration, reserve capacity, non-mitochondrial respiration, and coupling efficiency) were calculated and are shown in Fig 3. Groups were compared using a two-way ANOVA with LSD test and post-hoc Bonferroni correction for multiple comparisons.

In both fresh and frozen PBMCs, after Bonferroni correction, statistically significant differences between CFS and control patients were observed in the same four parameters; basal respiration (p≤0.043), proton leak (p≤0.013), maximal respiration (p≤0.003), and reserve capacity (p≤0.012) (Fig 3).

When fresh and frozen PBMC samples from CFS patients were compared, basal respiration (p = 0.006), ATP production (p = 0.003), maximal respiration (p<0.001) and reserve capacity (p˂0.001) differed significantly (Fig 3). Similarly, in healthy controls, significant differences were seen in basal respiration (p = 0.008), ATP production (p = 0.047), maximal respiration (p<0.001) and reserve capacity (p<0.001) between fresh and frozen samples. The consistently significant decrease in basal respiration and maximal respiration in frozen samples compared to freshly isolated PBMCs is thought to be due to stress as a result of the freezing process.

### Effect of glucose concentration on PBMC OCR

**Fig 4:**
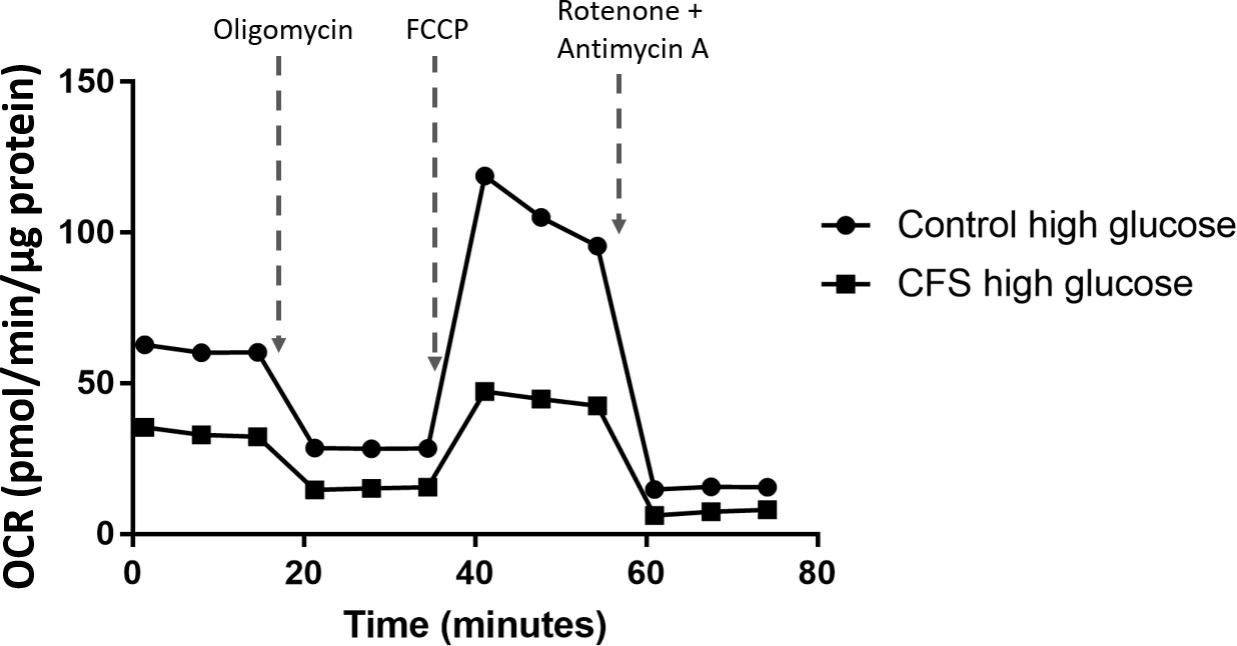
Example trace of a mitochondrial stress test performed in control and CFS PBMCs incubated for 24 hours in high (10mM) glucose.

Mitochondrial stress tests were also run in the presence of low (1mM) and high (10mM) glucose medium with the aim of seeing if changes in cellular ATP production could be detected between the two concentrations. It was hypothesised that PBMC incubation in low glucose conditions would cause the cells to be directed away from energy transduction via glycolysis and towards the OXPHOS pathway, causing an increase in mitochondrial respiration. This hypothesis predicts higher oxygen consumption rates in low glucose conditions compared with high glucose. Low glucose conditions were used to replicate a natural stressor (hypoglycaemia) that PBMCs may experience in *vivo* which is in contrast to the stress of freezing previously investigated, which is not experienced by PBMCs in *vivo*. An example trace of the mitochondrial stress test results are shown in Fig 4.

**Fig 5:**
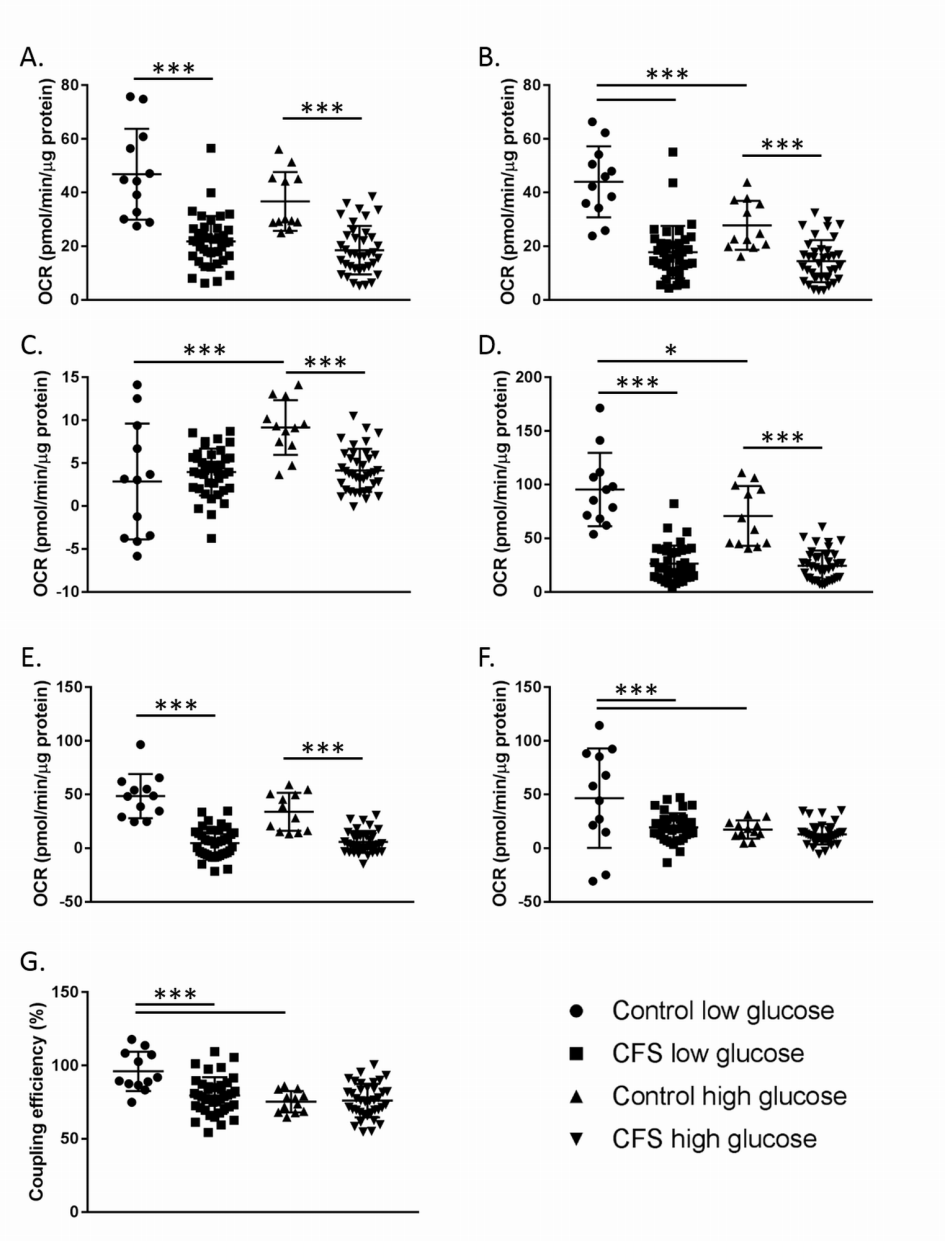
Results from a mitochondrial stress test conducted using CFS and control PBMCs incubated for 24 hours in low (1mM) and high (10mM) glucose. **A.** Basal respiration. **B.** ATP production. **C.** Proton leak. **D.** Maximal respiration. **E.** Reserve capacity. **F.** Non-mitochondrial respiration. **G.** Coupling efficiency. Control low glucose n = 12; CFS low glucose n = 39; Control high glucose n = 12; CFS high glucose n = 38. * denotes p ≤ 0.05; *** denotes p ≤ 0.005. Groups were compared using a two-way ANOVA with LSD test and post-hoc Bonferroni correction for multiple comparisons.

In low glucose, the CFS population had significantly lower results than the controls in six of the seven parameters measured; basal respiration (p<0.001), ATP production (p<0.001), maximal respiration (p<0.001), reserve capacity (p<0.001), non-mitochondrial respiration (p<0.001) and coupling efficiency (p<0.001) (Fig 5). When cells were incubated in high glucose CFS and control PBMCs differed in five of the seven parameters measured: basal respiration (p<0.001), ATP production (p<0.001), proton leak (p = 0.013), maximal respiration (p<0.001) and reserve capacity (p<0.001) (Fig 5).

The parameters which showed significant differences when results were compared from low and high glucose incubations were ATP production (p<0.001), proton leak (p<0.001), maximal respiration (p = 0.025), non-mitochondrial respiration (p = 0.004) and coupling efficiency (p<0.001) (Fig 5). However, these differences were only observed in the control cohort. Glucose concentration did not have any effect on the parameters within the CFS cohort.

Age of participants were correlated with maximal respiration (high glucose) using Pearson’s correlation. The results eliminate age (p = 0.217) as a confounding factor for mitochondrial function (Figure A S2 File). Student’s t-tests were conducted to see if sex of participants had an effect on maximal respiration (high glucose) (Figure B S2 File). There were no significant differences between female and male participants and maximal respiration in either the control (p = 0.630) or CFS (p = 0.862) cohorts, therefore sex can be eliminated as a confounding factor. Pearson’s correlation was used to investigate the relationship between the length of time PBMCs were frozen and cell viability as well as maximal respiration. Length of time of freezing did not correlate with cell viability (p = 0.100) or maximal respiration (p = 0.722) and can therefore be eliminated as a confounding factor (Figures C & D S2 File). The lack of correlation between length of time of freezing and maximal respiration shows that even short term freezing causes changes in OXPHOS function which proves that it is the freezing and reviving process that causes these changes as opposed to the length of time of freezing.

Results between plates for both the fresh/frozen and low/high glucose experiments were very consistent within each cohort due to the normalisation of data for protein concentration immediately after each assay.

### Glycolytic activity

**Fig 6:**
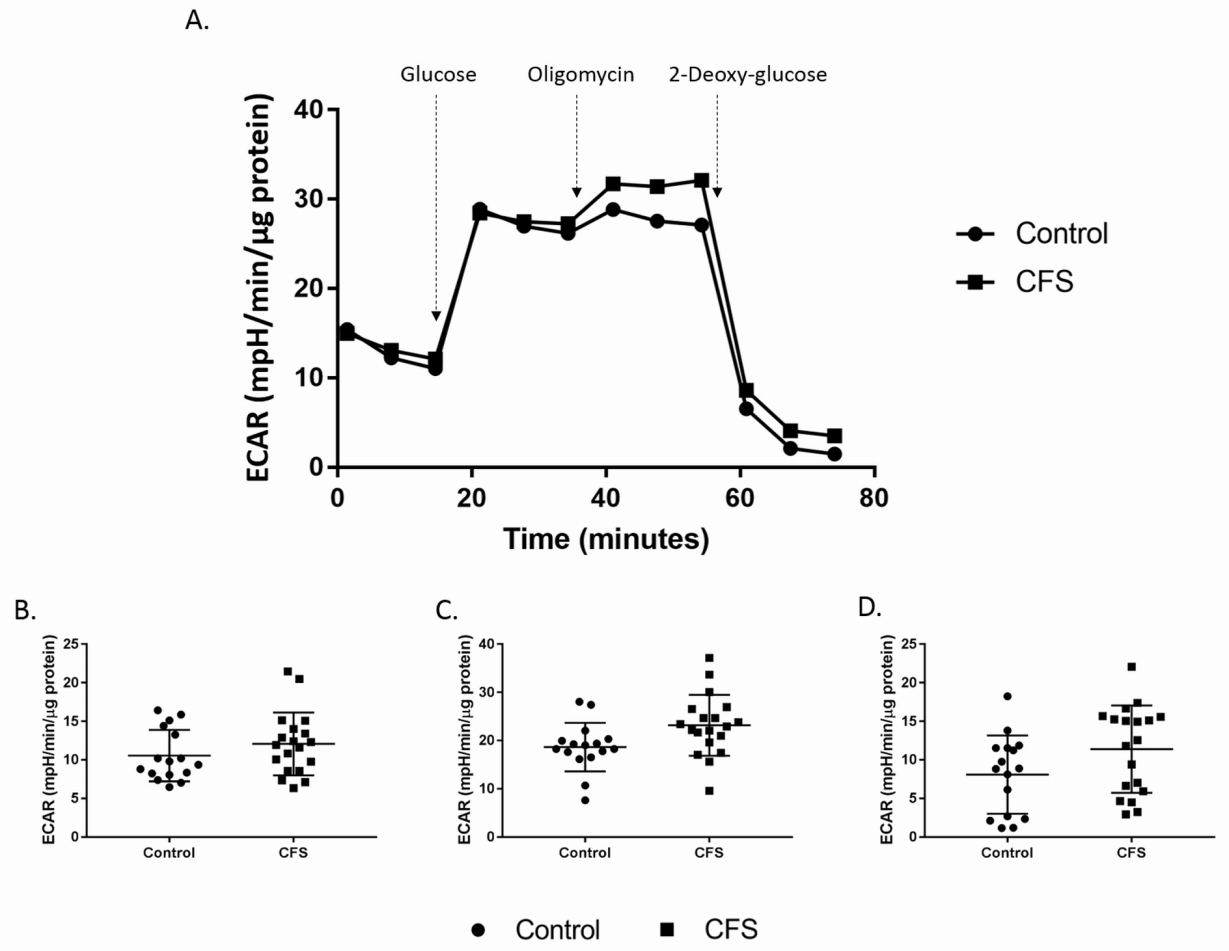
Assessment of cellular glycolytic function in CFS and control PBMCs. **A.** Results from a glycolysis stress test in PBMCs. **B.** Glycolysis, **C.** glycolytic capacity, and **D.** glycolytic reserve calculated from glycolysis stress test results. Control n = 16; CFS n = 19.

Results from a glycolysis stress test performed using PBMCs showed no differences in glycolysis between the patient and control cohorts (Fig 6). None of the five parameters calculated from the assay differed significantly between CFS and control samples (p≥0.075).

Cell viabilities of fresh and frozen samples were determined using Trypan Blue. The viabilities of fresh and frozen samples did not differ significantly and nor was there a significant difference between the viabilities of CFS and control samples, either fresh or frozen (p≥0.346), demonstrating the consistency of the cell viability between the two cohorts.

## Discussion

Despite large numbers of the population being affected, the causes of CFS remain unexplained. This is the first study to use case-control cohorts to investigate mitochondrial function in PBMCs in CFS. PBMCs were used for this preliminary study as it allows interrogation of potential abnormalities at a systemic level.

Significantly altered mitochondrial stress test parameters in the CFS group compared with the healthy control group (Fig 3 & Fig 5) suggests that CFS patients may have systemic abnormalities in energy transduction, particularly when isolated PBMCs are put under mitochondrial stress. Lower reserve capacity observed in CFS patients are indicative of the cells of patients performing closer to their capacity in normal conditions without stress than healthy controls. Lowered maximal respiration suggests that the PBMCs of CFS patients are not capable of the same levels of respiration as healthy controls. Results showed that CFS patients can only increase their respiratory capacity 47% (±37%) from baseline when maximally stimulated by FCCP, which is significantly (p<0.001) lower than the 98% (±32%) increase in respiratory capacity achieved by control PBMCs. The consistently lower maximal respiration in the CFS cohort shows the inability of CFS patients to respire to the same extent as the control cohort when cellular stress is applied. The lower reserve capacity suggests that when CFS PBMCs come under stress they are less able to increase their respiration rate to compensate for the increase in stress leaving patients unable to fulfil cellular energy demands. Similar abnormalities in reserve capacity are thought to contribute to a range of other disease pathologies such as heart disease [49], neurodegenerative diseases in aging [50], and smooth muscle cell death [51].

The freezing process had a significant impact on the cellular bioenergetics of PBMCs in both the control and CFS cohorts (Fig 3). Four of the parameters investigated showed significant differences between the fresh and frozen samples in both cohorts; basal respiration, ATP production, maximal respiration, and reserve capacity. Results showed that the freezing process affected both cohorts similarly, causing decreases in each of the aforementioned parameters. Freezing of PBMCs can cause numerous cellular changes which could account for the decrease in OCR recorded in these experiments. Potential changes caused by freezing include oxidative damage, a loss of membrane integrity due to the formation of ice crystals and changes in ion homeostasis [52].

Although freezing was shown to impact certain parameters, the same parameters were seen to be significantly different between the control and CFS cohorts in both fresh and frozen samples. This demonstrates that even though the absolute values of some of the parameters are affected by the freezing process, the pattern can be seen in both cohorts and therefore either fresh or frozen samples can be used to detect differences between control and CFS cohorts. For future studies wishing to look at absolute OCR values then fresh samples should be used, however, for other experiments, frozen samples are adequate to show the differences between the cohorts sufficiently. Frozen samples were used for the high and low glucose experiments due to sporadic sample collection over a relatively long time period, but this finding validates the use of frozen samples in these experiments and suggests that the freezing of samples does not affect the ability to compare cohorts. It should be noted that during early analysis with sample sizes ≤12, differences between the two cohorts were difficult to detect in frozen samples, therefore for smaller samples sizes the use of fresh samples would be advised to detect differences between cohorts.

It was hypothesised that PBMC incubation in low glucose conditions would cause the cells to be directed away from energy transduction via glycolysis and towards the OXPHOS pathway, causing an increase in mitochondrial respiration. This hypothesis predicts a higher basal respiration and maximal respiration in low glucose conditions. A higher maximal respiration in low glucose conditions was only observed in the control cohort but not the CFS cohort, while basal respiration did not increase significantly in either cohort in the low glucose conditions. Additionally, the control cohort increased ATP production, non-mitochondrial respiration and coupling efficiency, and decreased proton leak, in low glucose conditions. The CFS cohort showed no differences between low and high glucose conditions in any of the parameters. This may be due to the control cells being more adaptable to their environment and possessing an ability to increase their ATP production via mitochondrial respiration when required–something the CFS cells may not be able to do.

Four of the parameters were shown to differ between the control and CFS cohorts in both the low and high glucose concentrations; basal respiration, ATP production, maximal respiration, and reserve capacity. This demonstrates that the differences in these parameters between the two cohorts observed in Fig 3 could be reproduced in different glucose conditions and confirms the inability of the CFS PBMCs to increase their respiratory capacity to the extent that control PBMCs do both at baseline and when maximally stimulated by FCCP, even in low glucose conditions when the cells are pushed towards ATP production via mitochondrial respiration and away from glycolysis as an energy source. Non-mitochondrial respiration and coupling efficiency only differed between the control and CFS cohorts in low glucose conditions, while proton leak only differed between the two cohorts in high glucose conditions.

Coupling efficiency showed that ATP production only became more efficient in the control cohort when put under the stress of low glucose conditions, and not in the CFS cohort. Differences in coupling efficiency were seen between the control and CFS cohorts under low glucose conditions which suggests that the CFS cells are already performing at their maximum rate of efficiency for producing ATP which control cells are able to increase the efficiency at which they produce ATP when required e.g. under low glucose conditions.

Throughout the analysis process statistical analysis was conservative, using Bonferroni correction to correct for multiple comparisons introduced by looking at multiple parameters of respiration, as such multiple comparisons increase the risk of type I error. The statistically significant results shown in Fig 3 and Fig 5 indicate strong data, robust to statistical correction, despite the relatively small sample sizes used.

These experiments have helped to identify the direction future research into cellular bioenergetics in CFS should take by detecting differences in some, but not all, parameters of mitochondrial function. Differences in mitochondrial parameters shows the inability of CFS PBMCs to utilise the OXPHOS pathway to produce energy to the same extent as control PBMCs both in baseline conditions and when forced to maximally respire. The consistently significant differences between the two cohorts in basal respiration and maximal respiration demonstrates their importance as potential markers for CFS, a finding which could be utilised in directing future research into mitochondrial dysfunction in CFS.

Contrary to previous literature [15, 28] which suggested that abnormalities in PBMC ATP levels may be caused by glycolysis, results from the glycolysis stress test showed that glycolysis in CFS patients does not differ significantly from that of the non-disease cohort. This may be due to the relatively small sample sizes used in this study. The combination of the detection of significant differences in OXPHOS alongside the lack of detectable differences in glycolysis has potentially uncovered a previously unknown phenotype of CFS PBMCs, although larger samples sizes will be required in order for this to be confirmed. While the primary limitation of this study was sample size which resulted in underpowered analysis, particularly for the freshly isolated control cohort, it should be noted that the data presented here shows only preliminary findings. Higher sample sizes and longitudinal sample collection will be sought in the future to further validate the results obtained here, and potentially the power required to detect differences in other parameters. Another limitation of this study is the lack of characteristics of the CFS cohort. Demographic, anthropometric and symptom data would be useful to look for links with the mitochondrial abnormalities observed in this study. It would be interesting to analyse whether CFS symptoms, fatigue level in particular, correlated with any of the mitochondrial parameters measured. Additional experiments and techniques could be used to assess mitochondrial content and morphology, mitochondrial function in permeabilised cells and isolated mitochondria, and measures of mitochondrial membrane potential to further validate these results. Stratification of PBMCs using fluorescence activated cell sorting (FACS) is a technique that could be utilised to further sub-divide cell types that fall within of the category of PBMCs to identify which specific cell populations are responsible for the observed differences in OXPHOS. It is currently unknown whether factors such as recent activity and diet before blood collection has an effect on PBMC bioenergetics and this is an area that needs investigating. The use of sedentary controls and activity monitors in future studies would prove beneficial to show the effect of activity levels on PBMC bioenergetics. This study used PBMCs as a predictor for systemic mitochondrial function but other cell types could be investigated in future to investigate if the differences observed in this study can be seen in other tissue types in CFS patients.

The results of this study reflect the results from Hartman *et al*. in T2DM patients, showing a link between a different disease with fatigue as a core symptom and three OXPHOS parameters; basal respiration and maximal respiration [33]. This indicates that the PBMC bioenergetic abnormalities show a consistent link with fatigue, but whether the abnormalities occur as a result of the fatigue or are the cause of disease remains unknown.

This preliminary research has clearly shown statistically significant differences in the bioenergetic profile of PBMC’s derived from CFS patients when compared to non-diseased control cells. Importantly, these results do not establish whether differences in PBMC energy pathways are a cause or a consequence of CFS, however, this data clearly implies that these cells may play a role in the disease pathway. Further, the use of PBMCs may present a new and valuable model for the subsequent design of novel therapeutic approaches to the treatment of CFS.

## Supporting information

S1 File Shows the optimisation of oligomycin concentration, FCCP concentration and cell number for PBMCs on the XF^e^96 for performing mitochondrial stress tests. (DOCX)

S2 File Shows correlations for possible confounding factors. (DOCX)
