# Correction: Cellular bioenergetics is impaired in patients with chronic fatigue syndrome

**DOI:** 10.1371/journal.pone.0192817

**Published:** 2018-02-08

**Authors:** Cara Tomas, Audrey Brown, Victoria Strassheim, Joanna L. Elson, Julia Newton, Philip Manning

The fourth author’s name is spelled incorrectly. The correct name is: Joanna L. Elson.

The corrected citation is: Tomas C, Brown A, Strassheim V, Elson JL, Newton J, Manning P (2017) Cellular bioenergetics is impaired in patients with chronic fatigue syndrome. PLoS ONE 12(10): e0186802. https://doi.org/10.1371/journal.pone.0186802
