# Supplementary material for: Cellular bioenergetics is impaired in patients with chronic fatigue syndrome
Source: PLoS One. 2017 Oct 24;12(10):e0186802. doi: 10.1371/journal.pone.0186802 (PMC5655451; doi:10.1371/journal.pone.0186802)
Supplement: S1 File — (DOCX) [file pone.0186802.s001.docx]

S1. Seahorse optimisation in PBMCs


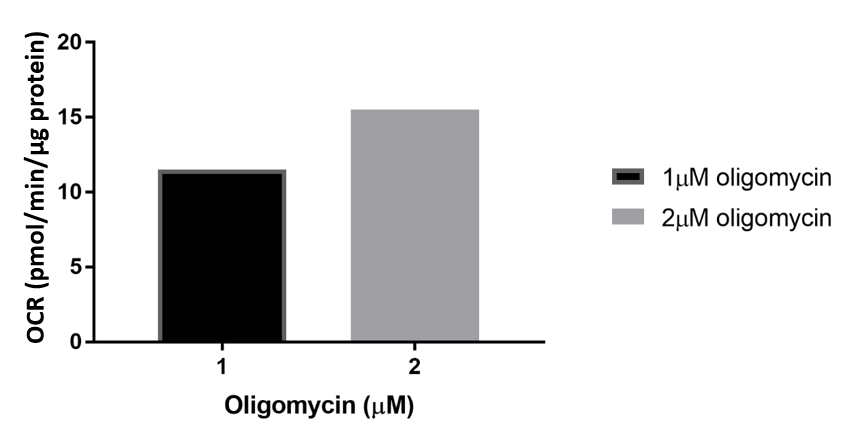


**Figure A. Oligomycin concentration optimisation in PBMCs**. Oligomycin was optimised using two concentrations of oligomycin – 1µM and 2µM. The typical concentration of oligomycin used in mitochondrial stress tests is 1µM. This optimisation also deemed 1µM to be the most appropriate oligomycin concentration as it caused the OCR to be recorded.


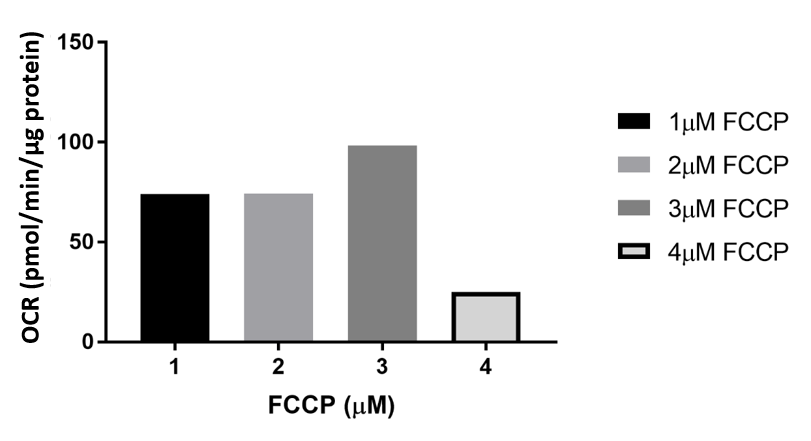


**Figure B. FCCP concentration optimisation in PBMCs.** FCCP was optimised using concentrations of FCCP ranging from 1-4µM. The optimal concentration is the lowest concentration at which maximal effect is seen. In this case 3µM was deemed to be the optimal concentration of FCCP to be used in PBMCs.


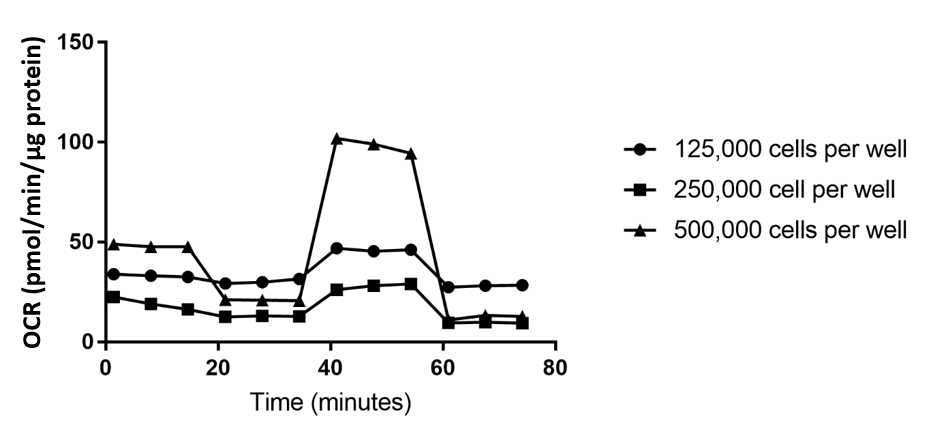


**Figure C. Cell number optimisation in PBMCs.** Cell number per well were optimised for use on the XF^e^96. The first cell number which achieved the typical shape of a mitochondrial stress test trace was 500,000 cells per well. At the lower cell numbers (125,000 and 250,000 cells per well) much lower OCRs were recorded and the drugs had a much lower effect on OCR therefore 500,000 cells per well was deemed the most appropriate for PBMCs on the XF^e^96.
