# Supplementary material for: Cellular bioenergetics is impaired in patients with chronic fatigue syndrome
Source: PLoS One. 2017 Oct 24;12(10):e0186802. doi: 10.1371/journal.pone.0186802 (PMC5655451; doi:10.1371/journal.pone.0186802)
Supplement: S2 File — (DOCX) [file pone.0186802.s002.docx]

S2. Possible confounding factor correlations


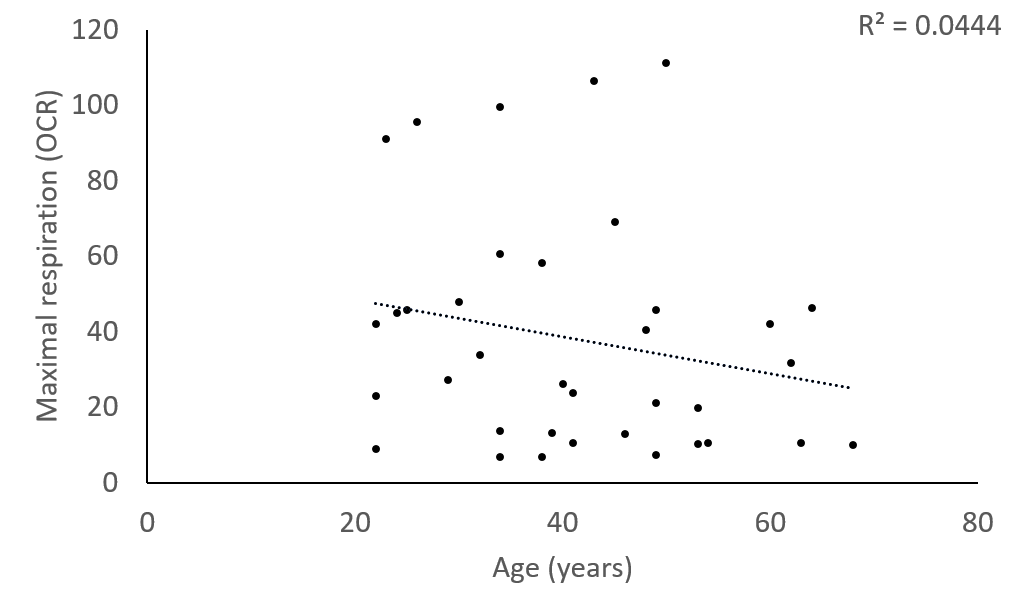


**Figure A. Age and maximal respiration correlation.** Age of participants was correlated with maximal respiration (Pearson’s correlation) for those participants whose samples were used in the high glucose experiments. There was no correlation between age and maximal respiration (P=0.270) therefore age can be eliminated as a confounding factor.


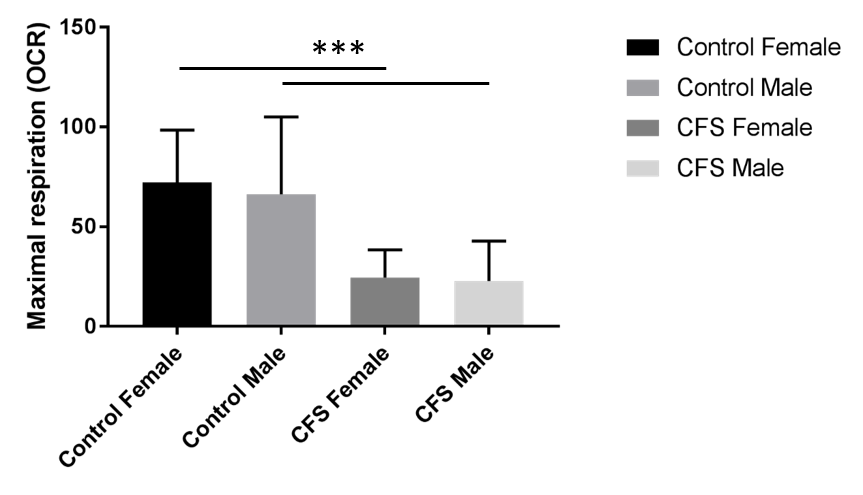


**Figure B. Relationship between sex and maximal respiration.** Student’s t-tests were conducted to see if sex of participants had an effect on maximal respiration (high glucose). There were no significant differences between female and male participants and maximal respiration in either the control (p=0.630) or CFS (p=0.862) cohorts, therefore sex can be eliminated as a confounding factor. As expected, there were significant differences between the control and CFS cohorts in both male (p=0.004) and female (p<0.001) participants. (*** denotes p<0.005).


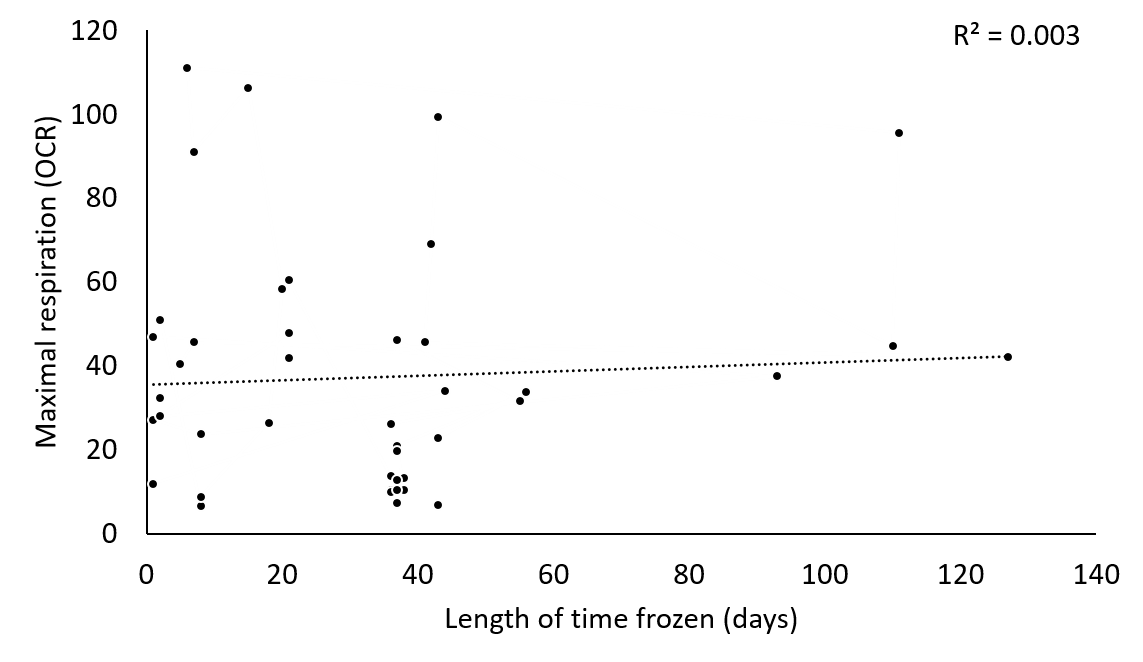


**Figure C. The correlation between maximal respiration and the length of times cells were frozen was determined.** Using Pearson’s correlation, maximal respiration (high glucose experiments) and the length of times cells were frozen were correlated and it was determined that length of time of freezing did not correlate with maximal respiration (p=0.645).


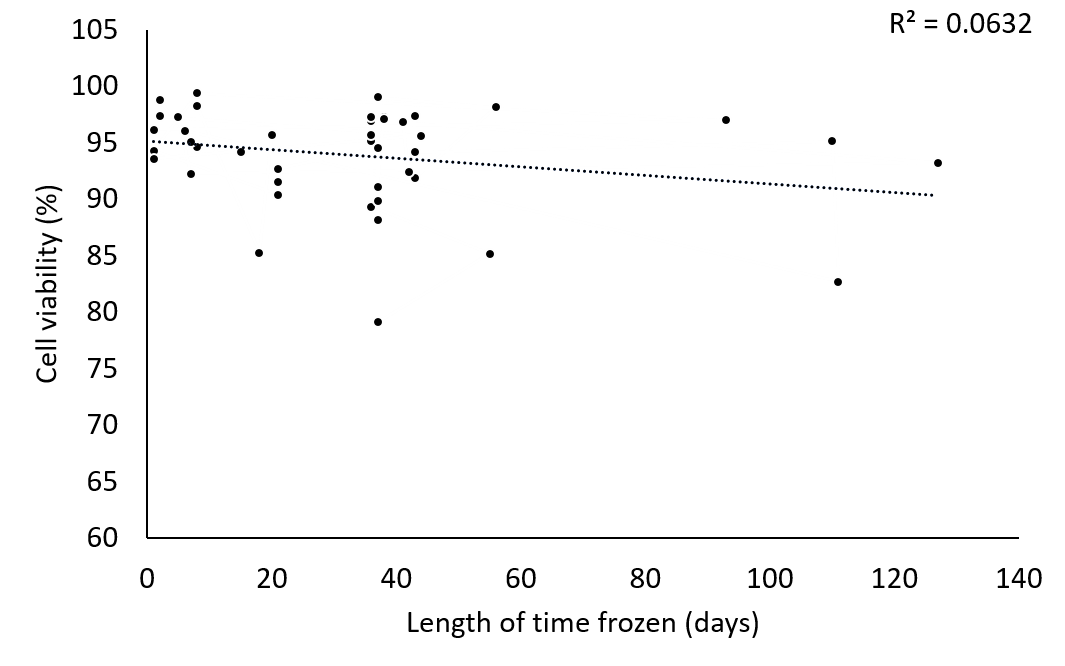


**Figure D. The correlation between cell viability and the length of times cells were frozen was determined.** Using Pearson’s correlation, cell viability after revival and the length of times cells were frozen were correlated. It was determined that length of time of freezing did not correlate with cell viability after revival (p=0.116). This result combined with that shown in S2-Fig2 s=eliminates length of time of freezing as a confounding factor.
